# Content Analysis of the Portrayal of Prescription Stimulants on TikTok

**DOI:** 10.1177/10870547251318834

**Published:** 2025-02-18

**Authors:** Benjamin Johnson, Tianze Sun, Leo Wu, Stella Seal, Daniel Stjepanovic, Gary Chan, Janni Leung

**Affiliations:** 1 The University of Queensland, Brisbane, Australia

**Keywords:** ADHD, psychostimulant, prescription stimulants

## Abstract

**Background:**

TikTok has become a significant influence on public health perceptions, especially regarding ADHD. With its expansive reach among younger demographics, TikTok content has the potential to shape public understanding and behaviors related to ADHD treatment. This study analyzed how prescription stimulants are depicted on TikTok to assess the potential influence of these portrayals on ADHD stimulant medication demand.

**Methods:**

We employed a snowball sampling strategy to collect 1,000 TikTok videos related to prescription stimulants. A systematic content analysis was conducted on a refined dataset of 548 videos, identifying primary categories related to prescription stimulant portrayals.

**Results:**

The videos, which predominantly featured young adults, mainly white and female, amassed an average of over 300,000 views per video. Our analysis uncovered four primary categories: Positive Effects, Negative Effects, Context of Use, and Systemic Challenges. Videos frequently depicted substantial improvements in daily functionality and emotional well-being attributed to prescription stimulants, with side effects presented as manageable. The context of use highlighted the broad applications of these medications in daily life, while systemic challenges focused on issues such as healthcare barriers, medication shortages, and stigma.

**Conclusion:**

TikTok users’ portrayal of prescription stimulants were predominantly positive, emphasizing improved lifestyles, which may influence medication demand similar to direct-to-consumer advertising, particularly among young women. The depiction of systemic barriers underscores the complexity of accessing treatment, which may disproportionately affect individuals with ADHD and impact treatment adherence, warranting further research into audience reception of this content.

## Key Points

• TikTok videos about prescription stimulants for ADHD largely emphasize positive effects, highlighting significant improvements in daily functioning and emotional well-being, while presenting side effects as manageable.
• The content is primarily created by young adult users, mostly white and female, with each video averaging over 300,000 views, indicating a substantial reach and potential influence on public perceptions, especially within younger demographics.
• Many videos discuss systemic issues related to ADHD treatment, including healthcare access barriers, medication shortages, and societal stigma, underscoring the complexities individuals face in obtaining and adhering to proper treatment.

## Introduction

In the landscape of modern communication, social media has become a pivotal junction where health perceptions are increasingly formed and influenced (Quintero Johnson et al., 2017). TikTok, with its more than a billion active users (Doyle, 2023), exemplifies this trend, offering a plethora of short-form videos that span the spectrum from entertainment to education. Its significant reach, especially among younger demographics (Statista, 2023), has established the platform as an influential source of mental health information, shaping how mental health conditions like ADHD are perceived by the public (Eagle & Ringland, 2023; K.Johnson, 2022).

TikTok’s portrayal of ADHD, often through relatable and viral content, may contribute to a simplification of medical conditions and an increase in self-diagnosis of mental disorders (Bippert, 2023). The hashtag #ADHD on TikTok has garnered over 32 billion views (Tiktok, 2024), yet only a fraction of this content is considered accurate and educational (Yeung et al., 2022). An influx of relatable stories about daily forgetfulness, procrastination, and other common issues has featured prominently on these platforms, influencing viewers to speculate about their mental health and potentially leading more people to believe they may have ADHD (Yeung et al., 2022).

This increased exposure and self-identification may be contributing to prevalence inflation, where increased awareness and discourse about mental health conditions lead not only to the recognition of previously undiagnosed cases but also to the labeling of milder distress or attentional difficulties as clinical conditions (Foulkes & Andrews, 2023). Concurrently, prescription rates for stimulant medications have surged over the last decade. From 2012 to 2021, stimulant dispensing increased by 45.5%, with an additional 8% rise in 2021 alone (Danielson et al., 2023; IQVIA, 2022). This growth has been primarily driven by adults, particularly women, while rates among children and adolescents have remained stable or declined (Board et al., 2020; Danielson et al., 2023; Olfson et al., 2013; Sibley et al., 2023).

The implications of such widespread engagement are multifaceted, with one being the nationwide shortage of ADHD stimulant medications in the United States, caused by unprecedented demand for ADHD treatment. Many point to the escalation of social media usage during the pandemic (Dixon, 2023), especially among those with attentional problems (Muzi et al., 2021; Werling et al., 2021) as a key catalyst for the increasing demand for these medications (Biggs, 2022; Bobby & Sandhu, 2023; Korducki, 2022). This is evidenced by a study of Twitter posts that revealed numerous tweets in which individuals discussed self-diagnosing and pursuing professional diagnosis after viewing ADHD content on TikTok (Gilmore et al., 2022).

Studies in health communication have shown that portrayals of substances on social media can significantly shape individual behaviors and attitudes, with positive representations potentially leading to an increased likelihood of use (Cohn et al., 2023; Rutherford et al., 2023). Additionally, positive depictions of prescription drugs in advertisements by pharmaceutical companies have been found to increase demand for these substances (Dave & Saffer, 2012). This suggests that TikTok’s depiction of prescription stimulants may also be contributing to the observed trends in ADHD treatment demand.

This issue is particularly important given the recent stimulant shortage in the United States, which has been partially attributed to increased demand for ADHD stimulant medications (Lovelace, 2024; NHS, 2024). Understanding how prescription stimulants are portrayed on widely consumed platforms like TikTok is crucial, as these portrayals may influence public perceptions and demand for these medications. Despite the potential impact, there is a significant lack of research on the current content of prescription stimulants online, especially on platforms with extensive reach among younger demographics.

Thus, by conducting a systematic content analysis of TikTok videos, we aimed to understand how prescription stimulants are portrayed and their potential influence on public perceptions and medication demand.

## Method

**Figure 1.**
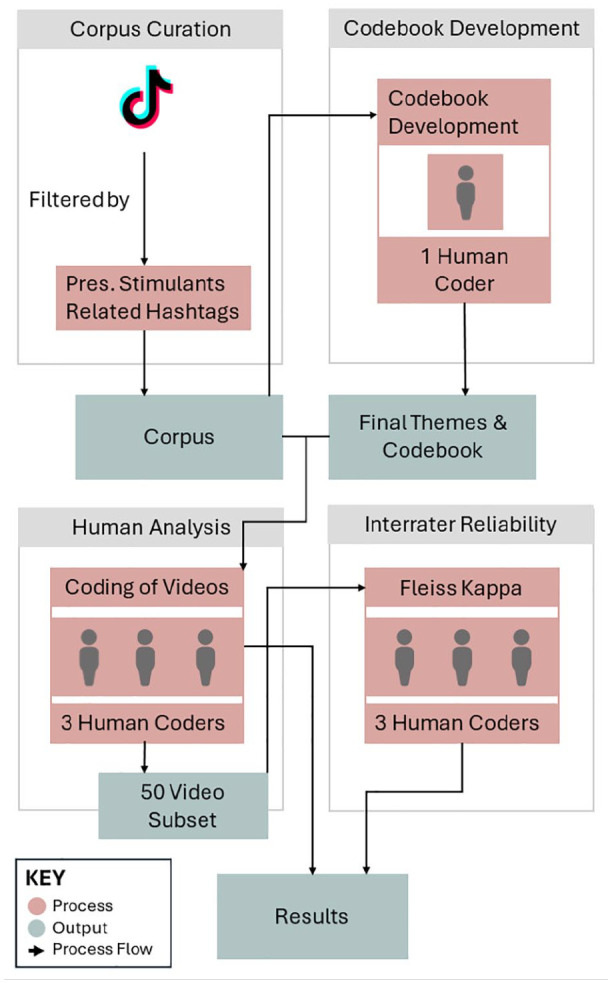
Process of data curation and content analysis.

The overall method was informed by research done previously on depictions of vaping (Sun et al., 2023) and cannabis (Rutherford et al., 2022) on TikTok. The full methodology can be seen in Figure 1.

In August 2023, our study applied a snowball sampling strategy to compile hashtags related to prescription stimulants on TikTok (Noy, 2008) to compile hashtags related to prescription stimulants on TikTok. This search was conducted on a newly created, TikTok set to 18+ to avoid content restrictions. We began by searching videos containing the initial hashtags #Vyvanse, #Adhdmedication, or #Adderall. From the most viewed videos under these initial hashtags, we noted additional hashtags that appeared relevant to prescription stimulants. These new hashtags were then searched individually, and further hashtags were recorded in the same way. This process was repeated with each new set of hashtags until no additional relevant hashtags were identified.

After compiling the full list, we manually screened each hashtag to confirm its relevance to prescription stimulants for ADHD and removed any unrelated ones. A minimum threshold of 10 million total views per hashtag was applied to ensure that only widely viewed content was included. This resulted in our final set of eleven hashtags: #Vyvanse, #Adhdmedication, #Adderall, #Adhdmeds, #Adderalshortage, #Elvanse, #Adderalcheck, #Vyvansesquad, #Concerta, #Vyvansetok, and #Adderaltiktok. We used a stratified sampling strategy, with the number of URLs extracted based on the relative number of views per hashtag to form a proportionally representative sample (Riffe et al., 2019).

The collected URLs underwent a screening process to eliminate duplicates and content not related to prescription stimulants. The final dataset was reduced to 548 videos after this process, with the exclusion of non-English content.

### Codebook Development

The codebook was derived from an initial random subset of 150 videos, selected to inform the development of our themes without relying on pre-established categories. This iterative approach was chosen to ensure that the analysis remained grounded in the data and reflective of the current discourse on prescription stimulants as portrayed on TikTok.

Our codebook consisted of demographic and content style variables of age, race, gender, genre, and type of stimulant use. Sociodemographic information was coded based on the researchers’ observations of visual and verbal cues within the videos. The primary categories were Positive Effects, Negative Effects, Context of Use, and Systemic Challenges, with their definitions detailed in Supplemental Table S2. Within the codebook, each primary category was further divided into subcategories to provide a detailed framework for analysis.

### Coding Process

The metrics of all videos (views, likes, and comments) were extracted by a single researcher in September 2023. Using the developed codebook, three researchers (BJ, SS, and LW) each coded approximately 200 videos, ensuring the entire dataset of 548 videos was covered. Videos were randomly assigned to coders to prevent overlap, meaning no video was double-coded during the primary coding phase.

To ensure consistent understanding of the codebook, researchers participated in weekly meetings during the coding phase. Any confusions or ambiguities encountered during coding were discussed and clarified collaboratively in these meetings.

Inter-rater reliability was assessed using Fleiss’ kappa on a random subset of 50 videos, which were independently triple-coded by all three researchers. Fleiss’ kappa values ranged from .679 to .908 across categories, indicating substantial to almost perfect agreement. The specific kappa values, along with corresponding *z*-scores and *p*-values for each theme, are provided in Table 1.

**Table 1.** Interrater Reliability Scores for Each Theme.

Statistic	Positive effects	Negative effects	Context of use	Systemic challenges
Fleiss kappa	0.791	0.863	0.679	0.797
*z*	10.3	11.2	8.98	10.5
*p*-value	<.001	<.001	<.001	<.001

### Ethical Considerations

To adhere to ethical standards and respect user privacy we only analyzed publicly available content. The data extraction process was conducted manually to comply with TikTok’s user guidelines (TikTok, 2023). Ethical clearance for this study was obtained from the relevant institutional review board (Application ID: 2023/HE002359), confirming adherence to the ethical standards for research involving social media content analysis.

## Results

The videos were highly viewed, averaging a total of 324,987 views per video. The videos we collected spanned from September 2019 to August 2023, capturing a comprehensive range of content trends and evolving discussions about prescription stimulants over time. The metrics of these videos were taken at the time of coding. The full metrics of these videos by theme, including views, likes, and comments can be seen in Table 2.

**Table 2.** Total and Average Metrics of TikTok Videos Included by Category.

Category	Average views	Average likes	Average comments
Positive effects	319,564	35,434	511
Negative effects	295,938	37,338	654
Context of use	387,852	60,092	638
Systematic challenges	295,331	36,013	1,077

Our analysis revealed the following demographics within the videos: 58% featured young adults, with the majority of individuals identified as white (82%), and predominantly female (70%). The portrayal of stimulant use was medical in 89% of the cases. Further details are available in Table 3.

**Table 3.** Content Characteristics of TikTok Videos.

Content	% (*n*)
Age	
Older adults	1.5 (8)
Middle age	34.7 (185)
Young adults	57.8 (308)
Adolescents	2.3 (12)
Cannot identify	3.8 (20)
Race	
White	82.2 (438)
Asian	5.1 (27)
Black	5.1 (27)
Latino	2.1 (11)
Other	1.3 (7)
Cannot identify	4.3 (23)
Gender	
Male	27.2 (145)
Female	69.6 (371)
Cannot identify	3.2 (17)
Genre	
Informational	12.4 (77)
Personal experience	61.6 (381)
Memes	26.5 (165)
Non-medical stimulant use	
Medical use	87.9 (474)
Non-medical use of own prescribed stimulant	5.0 (27)
Non-medical use of diverted prescribed stimulant	2.6 (14)
Not specified	4.5 (24)

**Figure 2.**
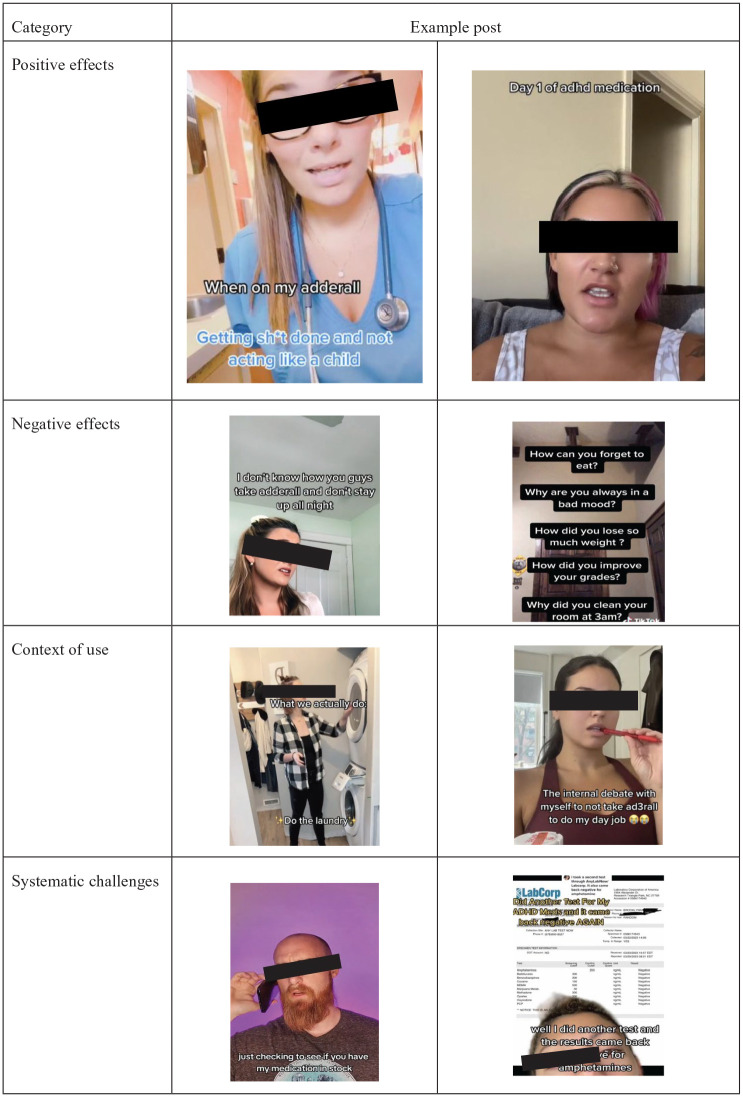
Examples of TikTok videos for each category.

The full frequencies and descriptions of our categories can be found in Table 4. Examples of each category can be seen in Figure 2.

**Table 4.** Full Frequencies and Descriptions of our Categories and Subcategories.

Category	Codebook description	*n*
*Positive effects*	This theme captured the perceived benefits and advantages that individuals experience while on ADHD stimulant medication.	223
Cognitive	Benefits such as increased calm mind, concentration, or attention.	110
Emotional or mood	Positive changes in mood, reduced anxiety	46
Motivation/productivity	Increased energy and drive to start and complete tasks	68
Weight loss	Favorably viewed weight reduction or appetite suppression attributed to the medication	22
Behavioral regulation	Improved self-control, reduced impulsivity, or more socially acceptable behavior	19
Social	Improved social interactions and communication skills or enhanced enjoyment and engagement in social activities.	18
General positive effect	Descriptions of improved quality of life or functioning without specific reference as to how	17
Other	Other positive effects not captured	15
*Negative effects*	This captured the negative side-effects that users described when taking prescription stimulants	187
Physical symptoms	Headaches, nausea, increased heart rate etc.	56
Appetite suppression	Challenges with eating and nutrition	46
Insomnia	Difficulties in falling asleep	31
Comedown	Negative effects experienced as the medication’s effects wear off, such as mood swings, increased anxiety, irritability, and cognitive fog	37
Active psychological issues	Negative emotional or psychological effects during the active effect of prescription stimulants	29
Tolerance	Encounters with tolerance, decreased efficacy over time, or feelings of medication “not working.”	19
Other	Other side-effects not captured	23
*Context of use*	This focuses on the various contexts and tasks in which individuals choose to use their stimulant medication. It explores how medication aids in specific areas of daily life, highlighting the functional and practical applications of medication use.	141
Work or study	Captures references to how medication aids in work-related tasks	49
Domestic efficiency	Relates to household chores, from cleaning to organizing, and how medication aids in accomplishing these	26
Hobbies and leisure	Instances where hobbies or leisure activities are more enjoyable or productive because of medication	16
General life	Improved daily task management	69
*Systemic challenges*	To capture broader, structural issues that individuals face when navigating healthcare systems in relation to stimulant medication. This theme seeks to understand the external challenges, beyond the direct effects of the medication itself, that may impact experience with ADHD treatment.	126
Healthcare barriers	Issues with healthcare access, insurance, and provider interactions.	48
Drug shortage	Difficulties accessing stimulant medication due to limited availability.	60
Medication authenticity	Doubts or concerns about the genuineness or efficacy of the medication	28
Stigma of medication use	Societal perceptions and stigma associated with stimulant medication.	22

### Positive Effects

Positive effects of prescription stimulants (*n* = 223) were frequently depicted in videos, illustrating the profound impacts these medications had on individuals’ lives. The content primarily featured users describing their personal experiences, often highlighting the initial stages of starting their medication. These narratives evoked strong emotions as individuals discussed the immediate, life-changing benefits they experienced, characterized by sudden and immense relief from symptoms. Videos focused on individuals initially starting on medication often omitted discussions of any negative effects and were overwhelmingly positive.

Many individuals also discussed their medication from a retrospective view. These typically provided a more balanced view of the positive and negative aspects of medication. These latter types of videos, however, often affirmed the ongoing benefits of the medications and their continued commitment to the treatment.

The most common improvements noted were cognitive (*n* = 110), with individuals reporting a stillness in thought that was markedly different from their usual chaotic and scattered minds. This enhanced individuals’ ability to concentrate and manage tasks effectively. Emotional benefits (*n* = 46) were also noted, including improved self-esteem and reduced anxiety.

Additionally, enhancements in motivation and productivity (*n* = 68) were reported, with individuals experiencing a greater capacity to initiate and sustain tasks without being distracted. Social benefits (*n* = 18) included diminished anxiety in social settings, facilitating more comfortable and engaging interactions. Weight loss (*n* = 22) emerged as another positive outcome, with individuals expressing satisfaction with their reduced appetite and subsequent weight reduction. Additionally, many provided a general endorsement of the medication (*n* = 17) reflecting on the broad positive impact on their lives.

### Context of Use

In numerous videos, individuals detailed the context they took prescription stimulants (*n* = 141) illustrating how these medications assist in various aspects of daily life. Typically, these videos showcased individuals taking their medication in the morning, followed by a depiction of their activities throughout the day. This approach helps to demonstrate the functional and practical applications of the medication in real time.

Other videos featured individuals who were already on their medication, focusing instead on the activities they engaged in while medicated. These narratives provided insight into the diverse motivations behind using the medication and the various life aspects that were enhanced by it. Notably, nearly all these videos were positive, predominantly showcasing the ways in which medication assisted in their lives, with very few videos depicting contexts where the medication had diminishing effects.

The most substantial subcategory, General Life (*n* = 69), captured the broad and versatile benefits of medication use, transcending specific activities like work or study (*n* = 49) and domestic efficiency (*n* = 26). Instead of limiting their narratives to particular tasks, many users shared how stimulants significantly enhanced their ability to manage daily activities as a whole. This was not confined to improving productivity in work or academic pursuits but extended to a general enhancement of life quality. A recurrent theme was the expression of a newfound sense of normalcy and capability, with many users marveling at their improved functioning in day-to-day life. Phrases like “I can’t believe this is how ‘normal’ people feel” underscored the transformative effect of medication, reflecting a comprehensive improvement in daily task management and personal well-being.

Additionally, Hobbies and Leisure (*n* = 16) highlighted how medication made leisure activities more enjoyable or productive, further supporting the notion that the benefits of stimulants permeate various facets of users’ lives.

### Negative Effects

Individuals also frequently discussed the negative side effects of prescription stimulants (*n* = 187). It is crucial to note the distinct tone used when discussing side effects compared to positive impacts. While many users acknowledged experiencing side effects, the majority did not express an intention to discontinue medication use or describe these side effects as significantly impairing their lives. Instead, individuals often framed them as inconveniences rather than major obstacles.

Physical symptoms such as headaches, nausea, and increased heart rate were noted (*n* = 56), highlighting the direct bodily impacts of these medications. Insomnia was another prevalent concern (*n* = 31), with many users detailing difficulties in falling asleep due to the stimulant properties of the medication, which adversely affected their sleep quality and daily functioning. Appetite suppression (*n* = 46) also emerged as a considerable challenge, with individuals reporting significant decreases in hunger and interest in food, leading to weight loss and changes in eating habits. This side effect was frequently discussed negatively, emphasizing the physical and lifestyle adjustments required by the medication.

The comedown phase (*n* = 37) was characterized by mood swings, increased anxiety, and cognitive cloudiness, often referred to as brain fog, marking a challenging transition as the medication’s effects waned.

Many individuals also experienced psychological issues (*n* = 29) during the medication’s active effect, reporting difficulties such as emotional blunting, depression, increased irritability, creativity loss and a “robotic” feeling. These issues represented deeper, more persistent challenges that impacted emotional health and personal identity during medication use.

### Systemic Challenges

Individuals prescribed ADHD stimulants shared the systemic challenges they faced in accessing and managing their medication (*n* = 126).

Healthcare barriers (*n* = 48) were a prominent concern. Users detailed difficulties in interactions with healthcare providers, where they felt their concerns regarding side effects and dose management were often dismissed.

Additionally, individuals described frustration at the healthcare system’s strict regulations on medication dispensation and the need for constant vigilance due to policy shifts and insurance changes. Individuals described how insurance policies would frequently only cover specific brands or generics and sometimes alter coverage without notice. This would also be a problem when individuals had to switch medication due to efficacy or availability and were put on medication, they did not have coverage for. Without proper insurance coverage, the cost of medication itself presented a substantial financial burden, often exceeding $200 USD.

The drug shortage (*n* = 50) emerged as a critical issue, with many individuals unable to access their medication. Individuals on the platform described widespread shortages, forcing them to undertake exhaustive searches for their medication, often without success. The resultant disruptions to treatment were profound, with some individuals having to halt their medication or switch to less effective alternatives.

Medication authenticity (*n* = 28) concerns were also significant, with users reporting changes in the effectiveness of their medication, leading to speculation about alterations in the formulation or the presence of placebos. Feedback loops of shared experiences reinforced these concerns, with some users conducting pharmacological tests that supported their suspicions about the medication’s legitimacy.

Stigma of medication use (*n* = 22) was another systemic challenge, with users encountering negative perceptions and advice to pursue alternative treatments like meditation instead of medication. The stigma was often perpetuated by the non-medical use of stimulant medication by individuals without ADHD, leading to a misrepresentation of those genuinely requiring medication for their condition. This societal stigma, coupled with the medical community’s cautious approach to prescribing stimulants due to abuse concerns, significantly impacted individuals’ experiences in accessing and using their medication.

## Discussion

The present study examined how prescription stimulants are portrayed in publicly available TikTok videos. Our analysis identified four themes reflecting how prescription stimulants were depicted: “Positive Effects,” “Negative Effects,” “Context of Use,” and “Systemic Barriers.” The vast reach of these videos, with an average of over 300,000 views and nearly 40,000 likes each, underscores their significant potential to influence public perceptions about prescription stimulants.

Our research identified that the videos predominantly depicted white individuals, reflecting the higher rates of ADHD diagnosis and medication treatment among white adults (Fairman et al., 2020). We also identified a predominance of young women among the content creators of prescription stimulant content on TikTok, despite ADHD being prescribed more often to males (Board et al., 2020; Sibley et al., 2023). This discrepancy suggests that young women may be more likely to share their experiences with stimulant medication on social media.

According to social identity theory, individuals are more influenced by and trust information from those similar to themselves, or their “ingroup” (Hogg, 2016). This means young women may be particularly receptive to these videos, potentially driving the rising demand for ADHD diagnosis and medication among this demographic (Board et al., 2020). Conversely, the increasing diagnosis of ADHD among young women could also be driving the creation and popularity of TikTok content related to prescription stimulants.

Additionally, the depiction of weight loss as a positive side effect might resonate strongly with young women, a group susceptible to body image pressures (Pan et al., 2023; Perloff, 2014; Tiggemann & Slater, 2013). This may amplify interest in prescription stimulants, aligning their use with societal beauty standards due to their ability to facilitate weight loss. Evidence shows that exposure to appearance-focused, weight loss or disordered eating behaviors can increase body image concerns and eating disorders (Dane & Bhatia, 2023). This should encourage the critical monitoring of content that exacerbates body image insecurities and suggests medical treatments for esthetic gains.

Both positive and negative aspects of stimulant use were discussed on TikTok. However, the narrative of videos overall was predominantly optimistic. Videos often featured substantial improvements in daily functioning across multiple domains of life and highlighted significant, immediate relief from symptoms. Additionally, individuals would often discuss domains in which medication improved their lives. This portrayal may substantially influence audience perceptions, potentially increasing the desirability and perceived effectiveness of prescription stimulants.

While the majority of users appeared to be describing the medical use of prescription stimulants, it is important to note that the benefits described may not be specific to individuals with ADHD, with stimulants found to enhance cognitive performance in individuals without ADHD (Bagot & Kaminer, 2014). Additionally, qualitative investigations into the non-medical use of stimulants reveal similar improvements in productivity and cognition to those described in the TikTok videos analyzed (Kerley et al., 2015; Vrecko, 2015). Stimulants are commonly used non-medically for academic purposes (Ford & Ong, 2014; Lucke et al., 2018). Therefore, if these videos influence individuals to take ADHD medications, the perceived benefits they experience and their alignment with the positive narratives on TikTok may further reinforce their decision to use such medications.

The portrayal of stimulant medication on platforms like TikTok exhibits similarities with direct-to-consumer (DTC) pharmaceutical advertising. The content employs emotive and anecdotal narratives that focus on improved quality of life and well-being, similar to tactics used in DTC advertising (Dave & Saffer, 2012; Frosch et al., 2010). Both TikTok content and direct-to-consumer advertising disclose side effects, yet the primary focus in both contexts remains on the positive aspects, such as improved quality of life and enhanced functioning (Lacasse & Leo, 2009).

DTC advertising has been proven highly effective in driving consumer demand for pharmaceuticals (Dave & Saffer, 2012). By synthesizing these observations with existing research on the influence of substance portrayals, parallels can be drawn to the effects of DTC pharmaceutical advertisements, which have been shown to increase demand for advertised products (Donohue et al., 2007).

However, it is important to note the differing intentions and motivations behind these portrayals. Pharmaceutical advertisements are commercially driven and professionally produced to promote sales, whereas user-generated content on TikTok is often motivated by personal experiences and peer-to-peer sharing. Despite these differences, the fundamental mechanism of shaping behaviors through portrayal remains relevant across both contexts. Nevertheless, the overall impact of these depictions remains speculative, and further research is necessary to establish a more concrete understanding of how individuals interpret these portrayals and the influence they have on their behaviors.

The diverse range of positive effects reported by users, such as increased motivation, emotional well-being, and improved life management, illustrates the multifaceted nature of stimulant medications’ benefits. However, this diversity is not fully reflected in the current body of literature. While there is substantial research on the cognitive benefits of these medications, such as improved attention and reduced impulsivity (Hupli, 2021), there is a paucity of studies that investigate the broader life-enhancing effects that users anecdotally report (Vrecko, 2013).

We discovered that individuals find navigating the ADHD medication system frustrating and inconvenient. There is limited research addressing the barriers and inconveniences caused by the regulation of these controlled substances, highlighting a crucial gap in the literature that needs further investigation.

The regulatory environment is designed to balance medication safety with patient access, yet it may inadvertently complicate access for those with legitimate needs. The strict bureaucratic procedures around prescription and dispensing, while necessary, can be particularly challenging for individuals with ADHD, who often struggle with executive functioning skills such as organization and planning (Antshel et al., 2013).

Furthermore, the recent stimulant shortage in the United States has significantly impacted individuals. This shortage is not only a supply issue but also a matter of public health, as it affects the stability of treatment for patients relying on these medications. We found that the resultant disruptions to treatment were profound, with some individuals having to halt their medication or switch to less effective alternatives. Our findings align with qualitative investigations that explore the lived experiences of individuals affected by the stimulant shortage, highlighting similar challenges in medication access, treatment disruptions, and significant impacts on daily functioning ( B.Johnson et al., 2024). This further underscores the extensive scope of the stimulant shortage and its profound impact on individuals’ lives, emphasizing the urgent need to address these shortages to safeguard the wellbeing of individuals with ADHD.

The continuous need for ADHD treatment, combined with the high cost of medications, imposes a substantial financial and logistical strain on families, which may affect job stability and overall quality of life. This underscores the critical need for healthcare policies that ensure accessible and affordable ADHD care, addressing the barriers that delay treatment and emphasizing the importance of supportive measures for individuals and families managing ADHD.

Moreover, striking a balance between facilitating access to necessary medication for genuine ADHD patients and discouraging over-diagnosis or prescription of stimulants to individuals without a clinical need is crucial. Public education campaigns led by health organizations can play a vital role in addressing misconceptions about ADHD and its treatment on social media. These campaigns should aim to inform the public about the potential side effects and adverse effects of unnecessary stimulant medication use, highlighting the importance of proper diagnosis and adherence to prescribed treatments.

The role of social media algorithms in promoting certain types of content cannot be overlooked. Platforms like TikTok use algorithms to suggest content to users, potentially amplifying content that receives more engagement, regardless of its accuracy or potential harm. This highlights the responsibility of social media platforms in moderating content to prevent the spread of misinformation and harmful narratives, especially those related to health and well-being.

It is crucial to identify the demographics and psychographics of the audience viewing this content to understand who is being targeted by these algorithms. A deeper understanding of the audience can help us more accurately speculate about the potential impact of these depictions. For instance, if young women are the primary viewers, the risk of harm is elevated due to the high prevalence of eating disorders in this group. One key limitation of our study is that sociodemographic information, such as age, race, and gender, was coded based on the researchers’ perceptions of the videos. This method introduces the potential for misclassification, as it may not align with how individuals identify themselves. Future studies could address this by incorporating self-reported demographic data or engaging with creators directly to verify sociodemographic details.

Additionally, knowing the demographics viewing this content can aid in tailoring public health messages and interventions more effectively to those most at risk. A significant limitation of our study is the lack of detailed information about the audience engaging with this content. Future research should investigate the viewer demographics to provide a clearer picture of the impact of social media depictions on public health and to ascertain whether these vulnerable groups are being reached.

## Conclusion

Videos frequently depicted substantial improvements in daily functionality and emotional well-being attributed to prescription stimulants, with side effects presented as manageable. This may significantly influence audience perceptions, particularly among similar demographics, potentially contributing to an increased demand for these medications.

Additionally, our research highlights systemic barriers that individuals face in accessing medication, pointing to a gap in the literature and suggesting a need for healthcare policy reform. The recent stimulant shortage in the United States further underscores the critical impact of supply chain issues on public health. Given the potential influence of TikTok and the systemic challenges identified, there is an imperative for further empirical research to inform evidence-based healthcare policies and communication strategies that can effectively support individuals with ADHD and their families.

## Supplemental Material

sj-docx-1-jad-10.1177_10870547251318834 – Supplemental material for Content Analysis of the Portrayal of Prescription Stimulants on TikTok Supplemental material, sj-docx-1-jad-10.1177_10870547251318834 for Content Analysis of the Portrayal of Prescription Stimulants on TikTok by Benjamin Johnson, Tianze Sun, Leo Wu, Stella Seal, Daniel Stjepanovic, Gary Chan and Janni Leung in Journal of Attention Disorders
