# Supplementary material for: Content Analysis of the Portrayal of Prescription Stimulants on TikTok
Source: J Atten Disord. 2025 Feb 18;29(8):617–27. doi: 10.1177/10870547251318834 (PMC12064856; doi:10.1177/10870547251318834)
Supplement: sj-docx-1-jad-10.1177_10870547251318834 – Supplemental material for Content Analysis of the Portrayal of Prescription Stimulants on TikTok [file sj-docx-1-jad-10.1177_10870547251318834.docx]

**Table S1.** Hashtags and Included Videos

| *Name (#)* | Total views as searched in 28/10/23 | % | Required number of URLs to scrape | Actual number of videos included in study |
| --- | --- | --- | --- | --- |
| Vyvanse | 136,200,000 | 21.20% | 212 | 141 |
| Adhdmedication | 132,000,000 | 20.55% | 205 | 133 |
| Adderall | 120,000,000 | 18.68% | 187 | 86 |
| Adhdmeds | 106,700,000 | 16.61% | 166 | 72 |
| Adderalshortage | 33,800,000 | 5.26% | 53 | 26 |
| Elvanse | 31,000,000 | 4.83% | 48 | 16 |
| Adderalcheck | 28,200,000 | 4.39% | 44 | 31 |
| Vyvansesquad | 16,200,000 | 2.52% | 25 | 14 |
| Concerta | 14,400,000 | 2.24% | 22 | 7 |
| Vyvansetok | 13,500,000 | 2.10% | 21 | 11 |
| Adderaltiktok | 10,400,000 | 1.62% | 16 | 11 |

**Table S2. Codebook of themes and subthemes**

| **Theme** | **Definition/Purpose** | **Subthemes/Codes** |
| --- | --- | --- |
| Positive Effects | To gather and understand the perceived benefits and advantages that individuals experience while on ADHD medication. This theme illuminates how medication might enhance certain aspects of users' lives and well-being. | 1. Cognitive: Experiences of increased concentration or attention. 2. Emotional or mood: Calming effect, positive changes in mood, reduced anxiety  3. Motivation/Productivity: Increased energy and drive to start and complete both personal and professional tasks 4. Weight loss: Favourably viewed weight reduction or appetite suppression attributed to the medication.  5. Behavioural Regulation: Improved self-control, reduced impulsivity or more socially acceptable behaviour.  6. Social: Improved social interactions and communication skills or enhanced enjoyment and engagement in social activities. |
| Negative Effects | To identify and understand the potential downsides, side-effects, or challenges individuals face while on ADHD medication. It offers a balanced perspective, ensuring that not just the positive but also the potential pitfalls of medication are considered. | 1. Physical Symptoms: Headaches, nausea, increased heart rate  2. Appetite Suppression: Challenges with eating and nutrition 3. Insomnia: Difficulties in falling asleep 4. Comedown: Includes negative effects like mood-swings, anxiety, brain fog on comedown  5. Tolerance: Encounters with tolerance, decreased efficacy over time, or feelings of medication "not working." |
| Context of Use | This theme focuses on the various contexts and tasks in which individuals choose to use their ADHD medication. It explores how medication aids in specific areas of daily life, highlighting the functional and practical applications of medication use. | 1. Work or study: Captures references to how medication aids in work-related tasks 2. Domestic Efficiency: Relates to household chores, from cleaning to organizing, and how medication aids in accomplishing these. 3. Hobbies and Leisure: Instances where hobbies or leisure activities are more enjoyable or productive because of medication. 4. General Life: Improved daily task management. |
| Systemic Challenges | To capture broader, structural issues that individuals face when navigating healthcare systems in relation to ADHD medication. This theme seeks to understand the external challenges, beyond the direct effects of the medication itself, that may impact an individual's experience with ADHD treatment. | 1. Healthcare barriers: Issues with healthcare access, insurance, and provider interactions. 2. Drug Shortage: Difficulties accessing ADHD medication due to limited availability. 3. Medication Authenticity: Doubts or concerns about the genuineness or efficacy of the medication.  4. Stigma of Medication Use: Societal perceptions and stigma associated with ADHD medication. |
